# Occupational Therapists' Perceived Confidence in the Management of Concussion: Implications for Occupational Therapy Education

**DOI:** 10.1155/2019/9245153

**Published:** 2019-02-24

**Authors:** Christina Finn

**Affiliations:** New York Institute of Technology, Northern Boulevard, P.O. Box 8000, Old Westbury, NY 11568-8000, USA

## Abstract

The purposes of this study were to examine occupational therapists' perceived confidence in the treatment and assessment of patients who have sustained concussion, to determine what factors are associated with higher versus lower levels of confidence, and to determine if concussion was a topic covered in occupational therapy curricula. This study utilized an electronic questionnaire sent out to occupational therapists in all areas of practice through social media, AOTA discussion forums, and through a continuing education company. Results indicate that there is variability in occupational therapists' perceived confidence in evaluation and treatment for individuals who have sustained concussion. Chi-square analysis indicates that higher levels of perceived confidence are associated with more clinical experience and greater amounts of continuing education in the area of concussion. Occupational therapists may benefit from additional training and education in the area of concussion.

## 1. Introduction: Concussion and Occupational Therapy

Concussion and the cumulative effects of repetitive head trauma have become growing health concerns both within the mainstream media and within the health care community [1, 2]. Although most concussions are thought to resolve within two weeks [3], there are some individuals who experience prolonged symptoms of concussion. These symptoms may include visual, vestibular, cognitive, emotional, and sleep disturbances [4], and recent literature suggests that these symptoms may interfere with an individual's ability to work, participate in school, and engage in sports [3, 5, 6].

Although there are varying types of approaches to the management of postconcussion syndrome, one of the main emerging themes in the literature is a graded return to activity following the initial injury [7–9]. Graded return to activity recommendations such as return to play and return to learn currently recommend initial return to activity at subsymptom threshold, meaning individuals should limit or terminate activity that worsens symptoms [3]. However, participation in activity without exacerbation of symptoms may require modification of the task or environment as well as an internal awareness of what may precipitate symptoms, which can be challenging for many individuals, suggesting the need for a balanced return to activity. A balanced approach to activity that carefully considers the interaction between person, task, and environment is congruent with the foundations of the profession of occupational therapy, suggesting the significant relevance of occupational therapy for this particular diagnostic population.

Despite the relevance and valuable role of occupational therapy in the management of concussion, there is limited literature related to occupational therapy and concussion. A study exploring occupational therapists' perceived confidence related to concussion management may help to determine if occupational therapists possess the perceived confidence to manage concussion cases and to determine factors that contribute to greater levels of confidence. By determining factors that potentially increase confidence, occupational therapists may develop greater visibility in clinical practice and research related to this particular emerging area of practice. Although there have been various studies that have assessed the knowledge of health care professionals related to concussion [10–13], there are limited studies that explore the perceived level of confidence of health care professionals, particularly occupational therapists, regarding the management of concussion and symptoms related to concussion.

### 1.1. Purpose

The purposes of this study were to examine occupational therapists' perceived confidence in the treatment and assessment of patients with concussion, to determine what factors are associated with higher versus lower levels of confidence, and to determine if concussion was a topic covered in university occupational therapy curricula.

## 2. Methods

The electronic survey consisted of a self-administered questionnaire and cover letter outlining the objectives of the study. The questionnaire was divided into the following categories: (1) Demographics, (2) Clinical Confidence Level, (3) Treatment and Assessment Utilized for Concussion, (4) Referrals Received, and (5) Education and Training. The survey was sent first to four content specialists with extensive experience in cognitive, perceptual, and visual rehabilitation and/or concussion and was updated according to reviewer feedback. The survey was sent electronically through Survey Monkey© and was disseminated through various sources including social media and American Occupational Therapy Association discussion forums. In addition, survey invitations were sent through a continuing education agency that has multiple partnerships with hospitals and rehabilitation centers throughout the country. The survey was sent to practicing occupational therapists in all areas of practice throughout the country. Survey responses were anonymous with IP addresses blocked by turning on the IP restrictions feature, available through Survey Monkey.

### 2.1. Data Analysis

Data were first cleaned, coded, and entered into SPSS Statistics Version 23 (IBM Corp., Armonk, NY). Demographics were analyzed descriptively for frequency of years of experience, experience with concussion, and whether the therapist received concussion training in college (see Table 1). Chi-square testing was utilized to determine if differences in perceived levels of confidence existed based on years of experience, number of continuing education credits taken, and if concussion was included within the therapist's college curriculum. *P* values less than .05 were considered statistically significant. A Mann-Whitney *U* test was utilized to examine whether there was a difference in perceived confidence between those who see clients with concussion versus those who do not see clients with concussion.

## 3. Results

Responses were collected from 29 states within the United States, with a large response rate from the Northeast, particularly New York State and from a wide variety of clinical settings. Clinical experience ranged from less than 1 year to greater than 20 years. The highest level of education varied from bachelors to doctoral degrees, with the largest response rate for Master of Occupational Therapy (55.3%).

A total of 60% of respondents reported they had seen or are actively seeing individuals who have sustained concussions, while 39.7% reported that they did not work with individuals who had sustained concussions. Consistent with this response, 40.6% of respondents reported that they currently receive referrals to treat clients who have sustained concussion and 59.4% report that they do not currently receive referrals to treat individuals who have sustained concussions. This number may not be representative of all practicing occupational therapists but rather may reflect therapists that were inclined to respond to the survey based on interest. Respondents reported receiving referrals for visual skill retraining (55.6%), cognitive skill retraining (61.1%), return to school (38.9%), return to play (22.2%), and other, which included return to work and driving, vestibular, balance, home safety, and general evaluation and treatment.

131/153 participants responded to the question “Was concussion covered in your school's occupational therapy curriculum?” Although more than half of the respondents indicated that they have seen or are currently seeing individuals who have sustained concussions, only 24.4% reported that concussion was covered in their college curriculum, while 75.6% reported that concussion was not a topic covered. Further analysis was conducted utilizing chi-square testing and findings indicate that individuals with greater than 20 years of experience were less likely to report that concussion was covered in their school's curriculum, whereas respondents who had less than 5 years of experience were more likely to report that concussion was covered in their school's occupational therapy curriculum (*χ*^2^ = 60, *P* < .05).

**Figure 1:**
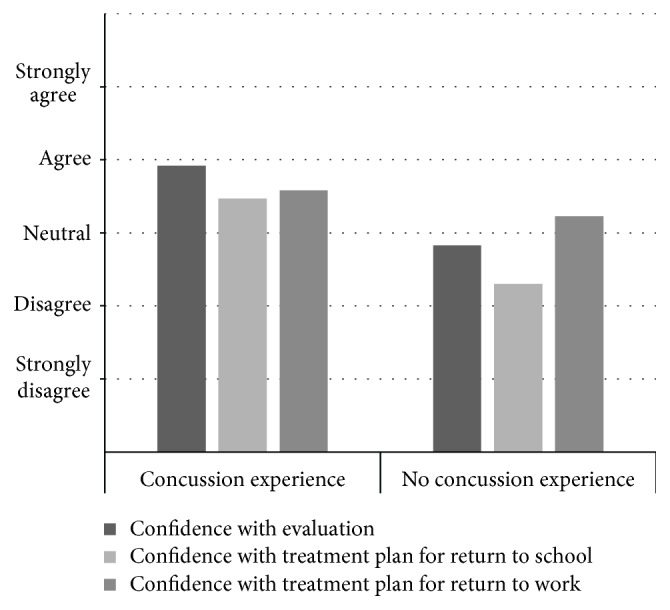
Perceived confidence and experience with concussion. Participants with and without clinical concussion experience reported their agreement with the following statements: “I am confident in evaluating a client with concussion, I am confident in developing a treatment plan for return to learn, and I am confident in developing a treatment plan for return to work.” ^∗^All values were statistically significant utilizing a Mann-Whitney *U* test (*P* < .01). *z* = −5.65 (I am confident evaluating a client with concussion). *z* = −3.27 (I am confident developing a treatment plan for return to school). *z* = −4.45 (I am confident developing a treatment plan for return to work).

Further analysis was conducted to determine what factors were associated with higher levels of perceived confidence. Differences between those who did not see clients with concussion and those who did see clients with concussion were examined with a Mann-Whitney *U* test and findings indicated that those who do not see concussion patients reported lower levels of perceived confidence in all areas of treatment and assessment (see Figure 1). Those who did not have experience with concussion also reported that they did not feel that they have adequate training/education to work with those who have sustained concussion in contrast to those who did have experience. Chi-square analysis was then conducted to determine what factors were associated with greater perceived levels of confidence for those who did have experience with concussion. Factors that were analyzed included years of experience, number of continuing education credits taken, and whether concussion was covered within occupational therapy curricula.

**Figure 2:**
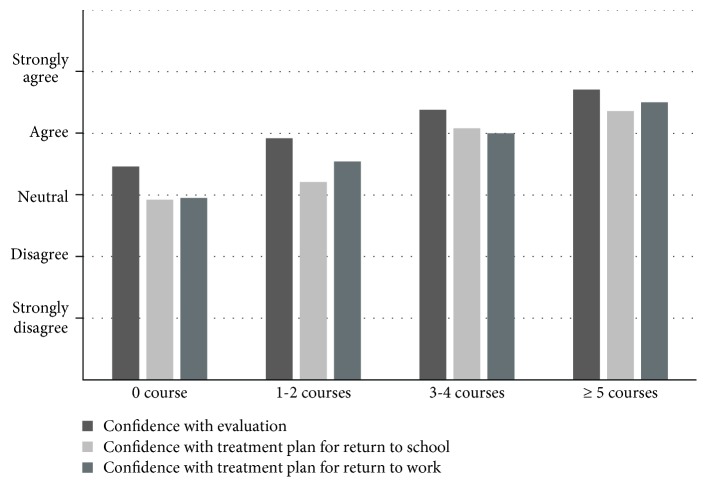
Perceived confidence and number of continuing education courses taken. Participants with and without clinical concussion experience reported their agreement with the following statements: “I am confident in evaluating a client with concussion, I am confident in developing a treatment plan for return to learn, and I am confident in developing a treatment plan for return to work.” ^∗^All values were statistically significant. *P* < .01. *χ*^2^ = 28.43 (I am confident evaluating a client with concussion). *χ*^2^ = 44.33 (I am confident developing a treatment plan for return to school). *χ*^2^ = 30.24 (I am confident developing a treatment plan for return to work).

For almost every question related to confidence, chi-square indicated that participants who had taken the most continuing education courses reported the greatest levels of confidence. In contrast, participants with the least number of continuing education courses had lower levels of perceived confidence (see Figure 2). No statistically significant findings were noted with regard to perceived confidence levels and whether the therapists reported if concussion was covered within their school's occupational therapy curriculum or year of experience.

## 4. Discussion

Results of this study indicate that occupational therapists report varying levels of perceived confidence with regard to evaluation and treatment of individuals who have sustained a concussion. In particular, reported confidence levels were lower for those not seeing clients with concussion and higher for those who have experience with concussion. The only factor that appeared to be consistently associated with higher perceived levels of confidence for all areas of concussion evaluation and treatment was the number of continuing education credits taken. Therapists who reported greater number of continuing education courses reported higher levels of perceived confidence in all areas while those with lower numbers of continuing education credits reported lower levels of perceived confidence. Years of clinical experience was not associated with higher levels of confidence in all areas of concussion evaluation and treatment.

Only 24.4% of respondents reported that concussion was covered in their school's occupational therapy curriculum, and data analysis indicates that these respondents were likely to have graduated within the last five years, suggesting that concussion maybe gradually becoming more prevalent in occupational therapy curricula. Concussion education within occupational therapy school was not associated with higher levels of perceived confidence in evaluation and treatment of individuals with concussion. However, the depth to which concussion was covered was not addressed in this survey.

To date, there is limited literature that examines perceived levels of confidence in the management of concussion, particularly within the profession of occupational therapy. However, there are studies that have examined knowledge levels in health care professionals and have found variability in the knowledge levels of health care professionals including emergency room physicians, physical therapists, and pediatricians [10–13]. One study found variability in knowledge of concussion for various health care professionals, with occupational therapists in particular, scoring lower than most other health care professionals, including physical therapists, speech therapists, psychologists, and athletic trainers on a concussion knowledge exam within one particular rehabilitation setting [13].

Although this study did not explicitly study the knowledge of occupational therapists, it did examine occupational therapists' perceived preparedness to work with individuals with concussion. The results indicate variability in responses related to perceived levels of confidence and with perceived levels of preparedness to treat a client with concussion, particularly for those without concussion experience. Although respondents may not reflect all occupational therapists, the results provide preliminary data on confidence related to concussion and suggests that many of the surveyed occupational therapists may not feel prepared or confident to provide occupational therapy for an individual who has sustained a concussion. One of the main findings that was associated with higher perceived levels of confidence was the number of continuing education courses taken. Thus, it appears that with further education and training, occupational therapists have the potential to develop greater levels of confidence in the area of concussion management. With greater levels of confidence, occupational therapists may be more inclined to develop and expand programs for individuals who have sustained concussion.

## 5. Limitations

Although the survey utilized in this study was piloted with experts in the area of concussion, the survey was not validated. In addition, since the survey was disseminated through various methods including social media, AOTA discussion forums, and through various rehabilitation centers throughout the country, an objective response rate was not obtained. Furthermore, although the sample reflected participants from a variety of geographic regions throughout the United States, the sample size was relatively small and does not reflect all practicing occupational therapists. The term concussion was not explicitly operationalized for participants. However, the term concussion was intentionally selected rather than mild traumatic brain injury as mild traumatic brain injury may be confused with other types of brain injuries. Lastly, occupational therapists who chose to respond to the questionnaire may have been interested in the topic of concussion.

## 6. Implications for Occupational Therapy Education and Practice

Current guidelines for the management of concussion call for a graduated return to activity at subsymptom threshold [3]. Occupational therapists have the unique potential to facilitate reengagement in activity following concussion at the appropriate level of challenge with task and environmental modifications. However, some occupational therapists may feel that they do not possess the confidence or training to work with clients who have sustained concussion as reflected by the results of this study. Greater confidence associated with more continuing education suggests the potential benefits of further education and training in the development of therapist confidence in this particular area of practice. Therefore, the incorporation of concussion into occupational therapy curricula may help the therapist to learn basic foundations of practice which may be further developed with continuing education courses. Concussion may be covered in existing physical rehabilitation courses that already cover traumatic brain injury and should address the role of occupational therapists in optimizing return to occupation with emphasis on applying current consensus guidelines for return to school and return to play.

- Concussion and concussion rehabilitation are developing and evolving areas of practice
- Occupational therapy can be a valuable service to those who have sustained concussion
- There is variability in occupational therapists' perceived confidence in evaluation and treatment of individuals who have sustained concussion
- Continuing education may be associated with greater levels of perceived confidence in the management of concussion
- Clinical experience with concussion may be associated with greater levels of perceived confidence in the management of concussion
- Occupational therapists may benefit from further education and training in the area of concussion

## Figures and Tables

**Table 1:** Therapist characteristics: clinical experience and education (*N* = 153).

Characteristic	Frequency (%)
Years practicing	
<1 year	3.3
1-5 years	22.4
6-10 years	13.8
11-19 years	27.6
20+ years	32.9
Highest level of education	
Bachelor's degree	21.1
Master of Occupational Therapy	55.3
Doctor of Occupational Therapy	13.8
Ph.D.	2.6
Other	7.2
Currently seeing or have seen concussion patients	
Yes	60.0
No	39.7
Concussion covered in OT curriculum *N* = 131	
Yes	24.4
No	75.6

## Data Availability

Data was collected through Survey Monkey and analyzed through SPSS. Results of the survey can be accessed through Survey Monkey.
